# Strains of a virtuoso: pacemaker infection and ventricular tachycardia in a violinist

**DOI:** 10.1186/s12872-025-04495-0

**Published:** 2025-01-23

**Authors:** Yuanguo Chen, Haibo Zhang, Qi Qiao, Lian Ma

**Affiliations:** 1 Department of Cardiovascular Medicine, Ya’an People’s Hospital, Ya’an, Sichuan Province 625200 China; 2 Department of Hematology and Oncology, Shenzhen Children’s Hospital of China Medical University, Shenzhen, 518038 China

**Keywords:** Pacemaker infection, Ventricular tachycardia, Lead extraction, Sick sinus syndrome, Occupational habits

## Abstract

**Purpose:**

Pacemaker-related infections are serious complications of cardiac implantable electronic devices (CIEDs). This case report aims to describe the occurrence of pacemaker pocket infection and recurrent ventricular tachycardia (VT) in a Chinese amateur violinist with sick sinus syndrome (SSS), and to explore the possible connection between occupational habits and the infection, as well as VT.

**Methods:**

A 76-year-old male violinist with a Biotronik Evia DR dual-chamber pacemaker presented with syncope and signs of a pacemaker pocket infection three years after implantation. Despite initial antibiotic treatment, the infection persisted with slightly elevated C-reactive protein (CRP) and negative cultures. The VT originated from the right ventricular outflow tract (RVOT), as confirmed by echocardiography and ECG findings. The infection was treated with debridement and extraction of the pacemaker and leads.

**Results:**

Debridement and extraction of the pacemaker and leads successfully resolved both the VT and the infection. The VT was likely linked to the infected lead, while the pacemaker infection was attributed to the patient’s violin playing, which caused mechanical stress and skin damage at the pacemaker site. Postoperative recovery was uneventful, with no recurrence of infection or arrhythmias at follow-up.

**Conclusion:**

This case highlights the importance of considering a patient’s occupational habits when selecting pacemaker pocket sites to prevent infections and complications. In this case, the patient’s violin playing likely contributed to mechanical stress at the pacemaker site, leading to infection. Early identification and appropriate management, including device removal, are crucial to prevent further complications.

## Case presentation

### Patient information

**Fig. 1:**
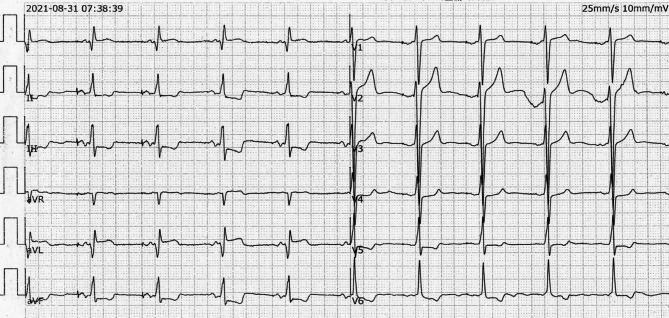
The electrocardiogram (ECG) post-pacemaker implantation demonstrates VAT pacing mode with an atrial pacing rate of 60 beats per minute. The ECG indicates effective atrial sensing, atrial triggering, and ventricular sensing without ventricular pacing

A 76-year-old male Chinese amateur violinist with a history of sick sinus syndrome received an Evia DR (Pro MRI) dual-chamber pacemaker manufactured by Biotronik three years prior. Post-implantation, the electrocardiogram (ECG) showed AAI pacing mode (Fig. 1).

### Symptoms and initial treatment

**Fig. 2:**
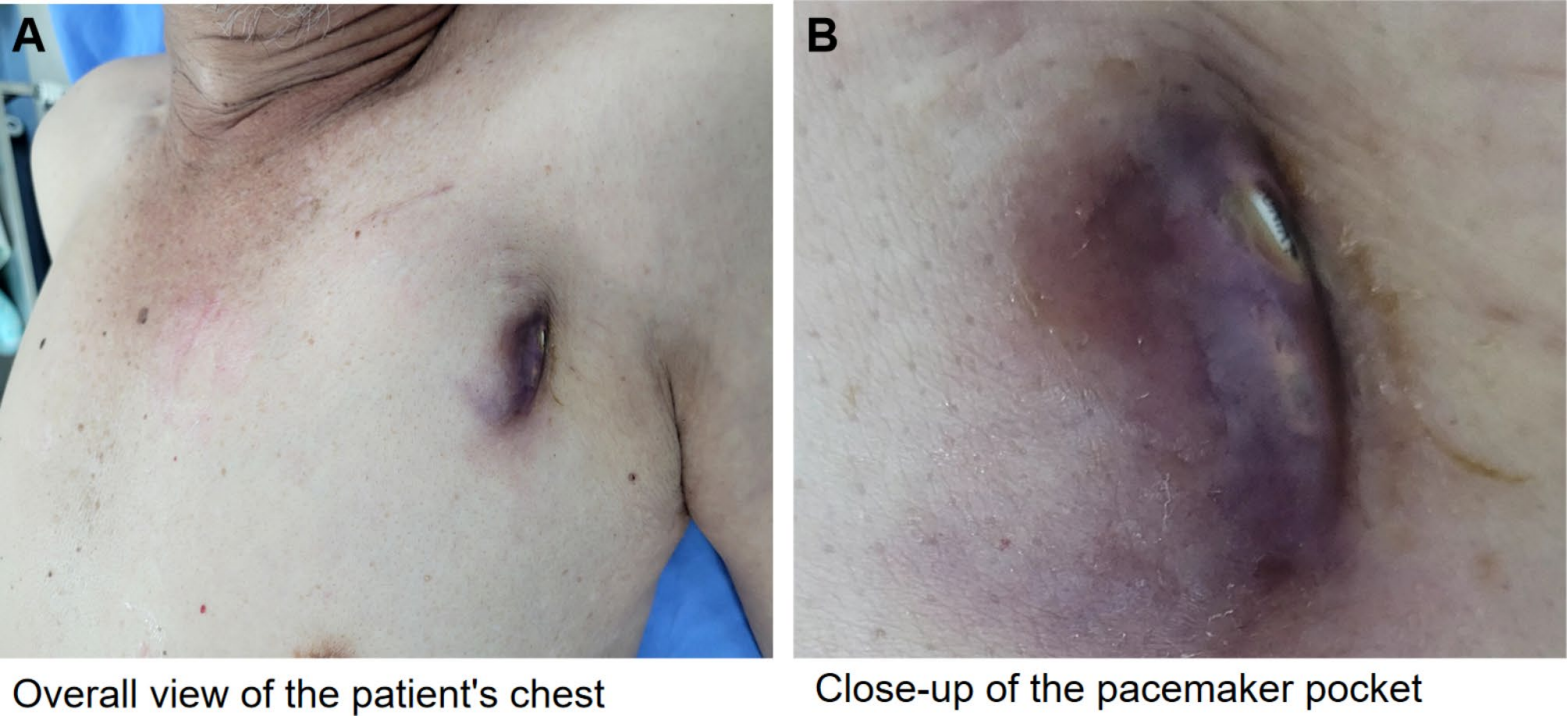
Panel A: An overall view of the patient’s chest, showing the location of the pacemaker. Panel B: A close-up of the pacemaker pocket. The images reveal a pacemaker pocket infection with exposed leads and visible skin necrosis around the area, indicating a severe infection

**Fig. 3:**
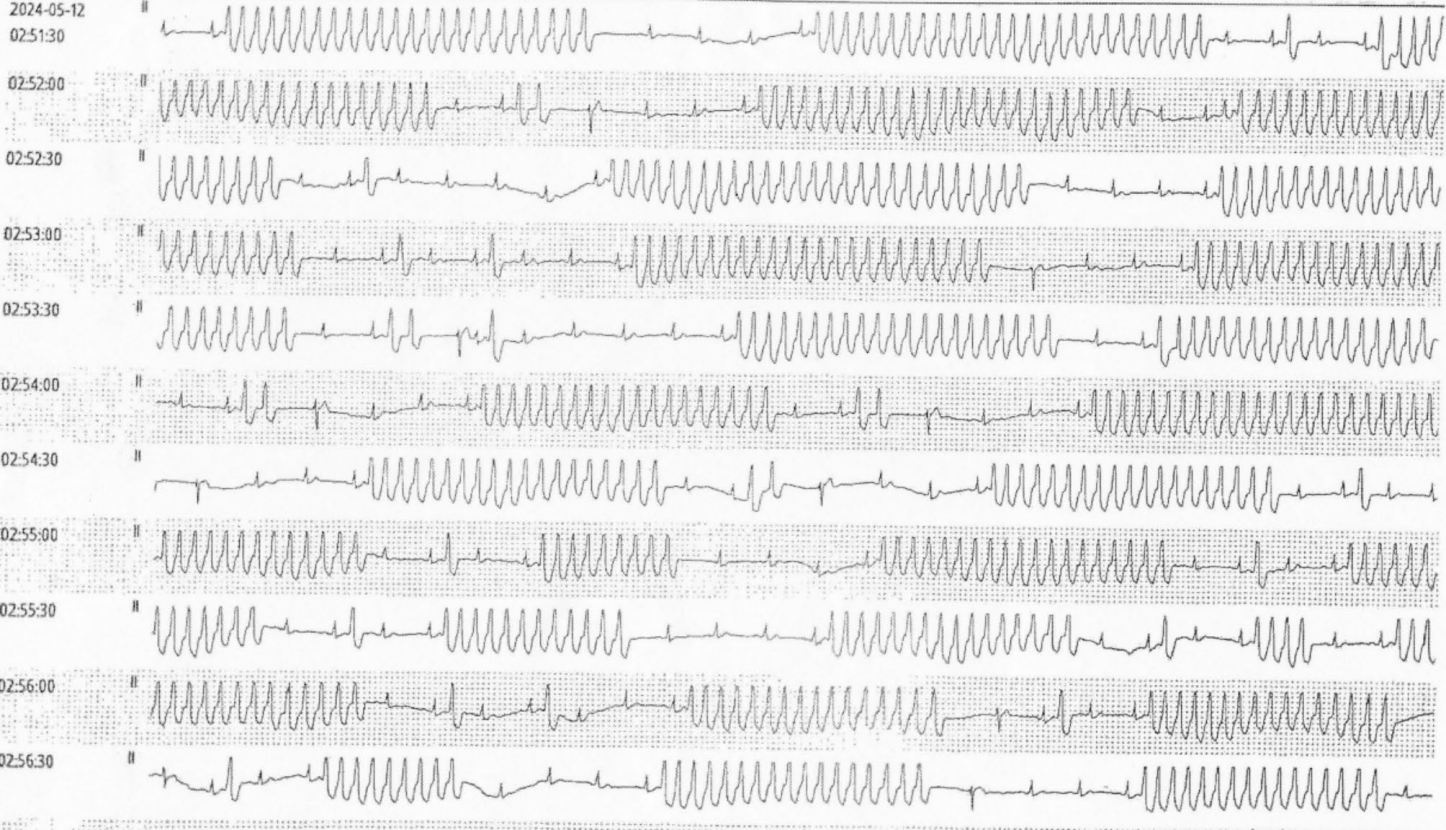
Recurrent ventricular tachycardia during the CCU hospitalization following pacemaker infection. The ECG tracings, recorded in standard lead II, highlight multiple episodes of ventricular tachycardia, indicating the severity of the arrhythmia associated with the pacemaker infection

**Fig. 4:**
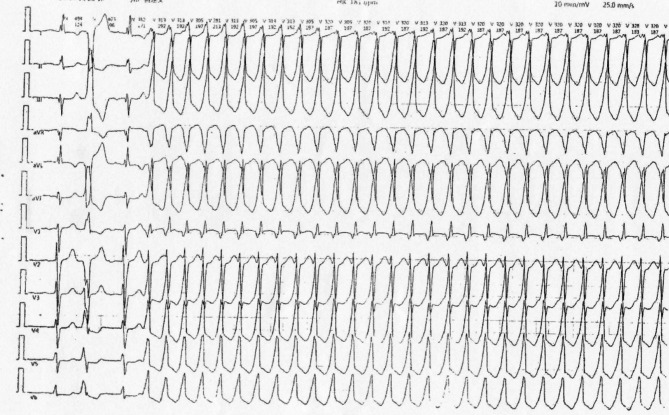
Standard 12-lead electrocardiogram (ECG) recording of a ventricular tachycardia (VT) episode in the patient. The VT has a rate of approximately 190 beats per minute. In lead I, the QRS complex displays an R-wave pattern. Leads II, III, and aVF show predominantly positive QRS complexes with the main deflection being upward. The precordial leads (V1-V6) indicate an early transition, characterized by the shift of the QRS complex to a predominantly positive direction earlier than expected, suggesting the VT may originate from a location other than the typical right ventricular outflow tract (RVOT)

**Fig. 5:**
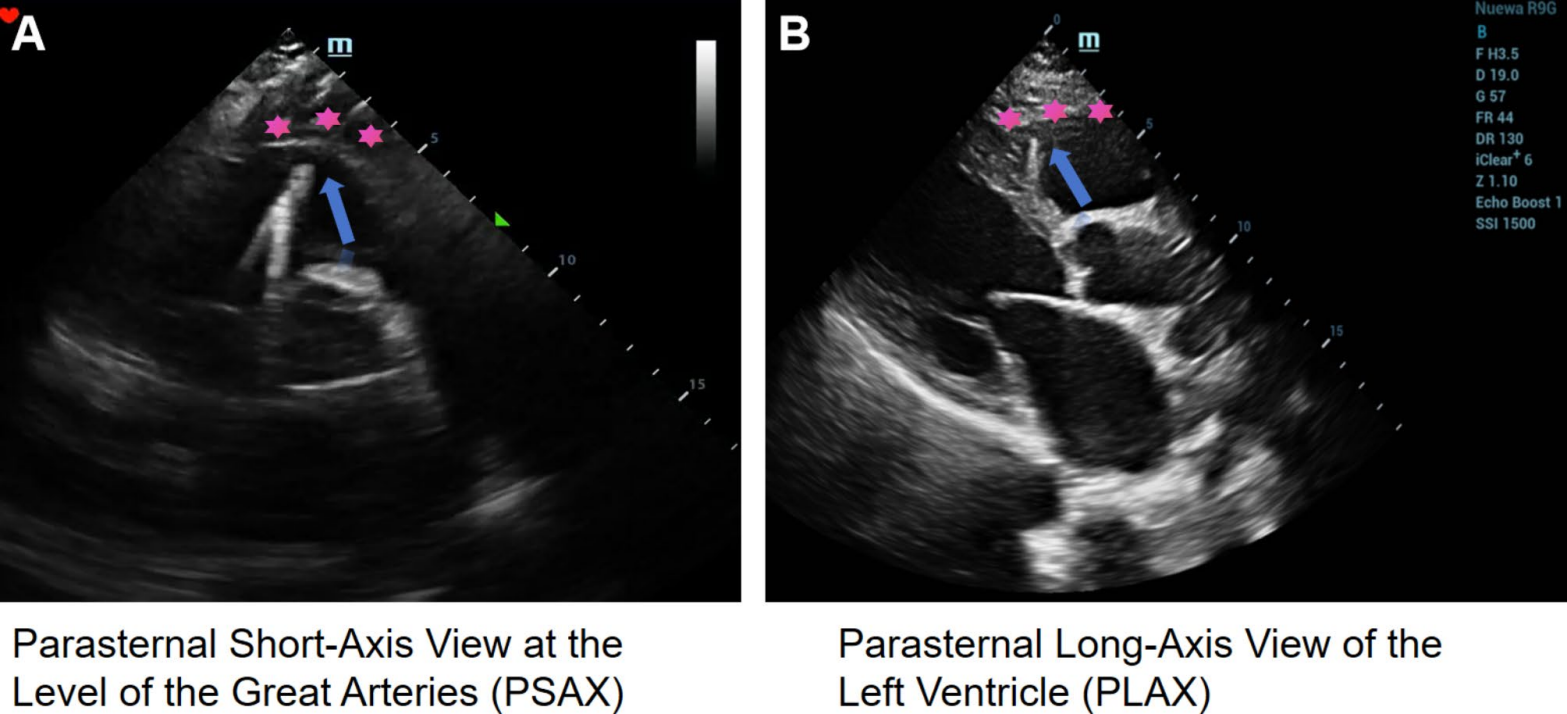
Panel A: Parasternal short-axis view of the great arteries. Panel B: Parasternal long-axis view of the left ventricle. In both images, the blue arrows indicate the ventricular lead, while the star symbol marks the free wall of the right ventricle. The images demonstrate that the right ventricular lead is positioned at the free wall of the right ventricle, which is identified as the origin of the ventricular tachycardia

**Fig. 6:**
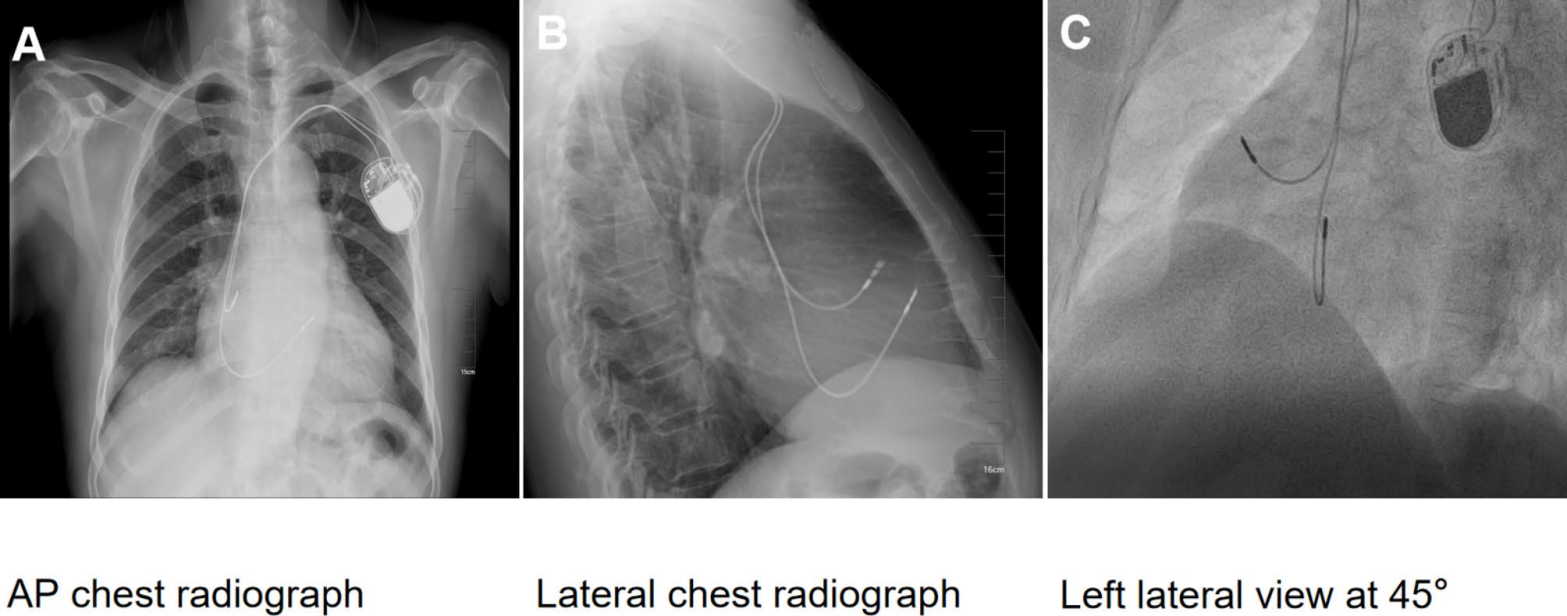
This figure is divided into three panels, illustrating the positioning of the pacemaker leads. Panel A: Anteroposterior (AP) chest radiograph. This image shows the overall position of the pacemaker leads within the chest. Panel B: Lateral chest radiograph. This side view provides additional detail on the spatial orientation of the leads. Panel C: Left anterior oblique (LAO) view at 45 degrees under digital subtraction angiography (DSA). This fluoroscopic image highlights the specific locations of the pacemaker leads. The images collectively indicate that the right atrial lead is positioned in the right atrial appendage, while the right ventricular lead is situated in the right ventricular outflow tract, leaning towards the free wall rather than the interventricular septum. These details are critical for understanding the lead placement and potential implications for cardiac function and arrhythmia management

Three years after implantation, the patient presented with syncope, localized redness, and pain due to pacemaker pocket infection and exposure under the left clavicle. Despite antibiotic treatment at a local hospital, the infection persisted (Fig. 2). Laboratory tests showed slightly elevated CRP but normal white blood cell count and other parameters. Blood cultures and pocket discharge cultures were negative. The patient presented with localized redness, pain, and syncope. ECG indicated recurrent VT (Figs. 3 and 4). Laboratory tests showed normal white blood cell count, liver and kidney function, electrolytes, and coagulation profiles. CRP was slightly elevated. Initial diagnosis was pacemaker pocket infection. Echocardiography (Fig. 5) and chest X-ray (Fig. 6) confirmed that the ventricular lead was positioned at the free wall of the right ventricular outflow tract, where the VT originated.

### Interventional procedure

**Fig. 7:**
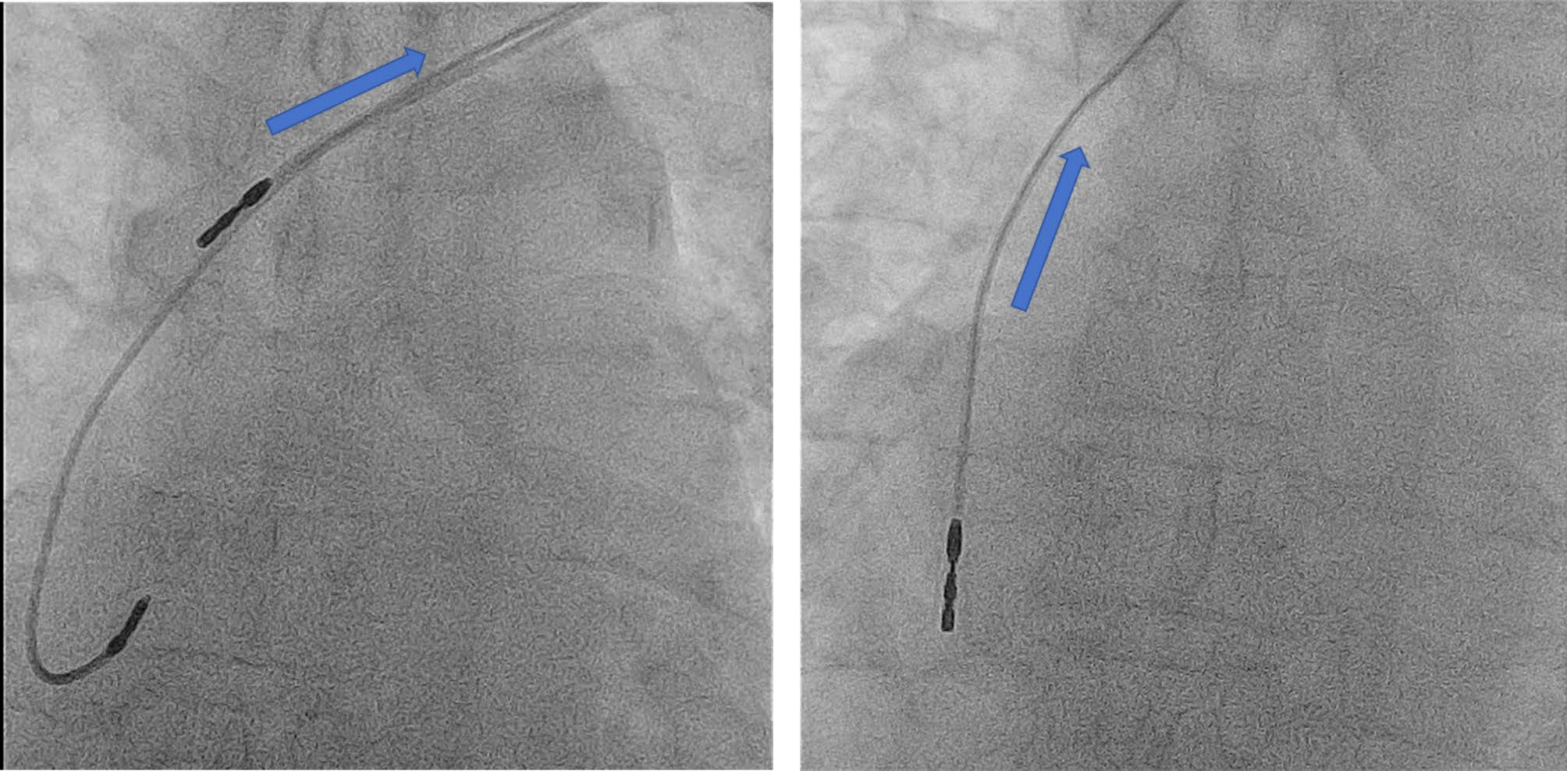
Panel A: Extraction process of the right ventricular pacing lead. The lead is unscrewed from the myocardial tissue by rotating it counterclockwise and then gently pulled out of the body. Panel B: Extraction process of the right atrial pacing lead. After the right ventricular lead has been removed, the right atrial lead is similarly rotated counterclockwise to disengage it from the atrial myocardial tissue and then carefully pulled out of the body. The blue arrows in both panels indicate the direction in which the leads are being pulled out of the body

Debridement and percutaneous extraction of the pacemaker and leads were performed. The extraction process involved counterclockwise rotation to detach the leads from myocardial tissue (Fig. 7). During the procedure, VT occurred but was managed successfully. Post-procedure, the patient received broad-spectrum antibiotics.

### Outcome

**Fig. 8:**
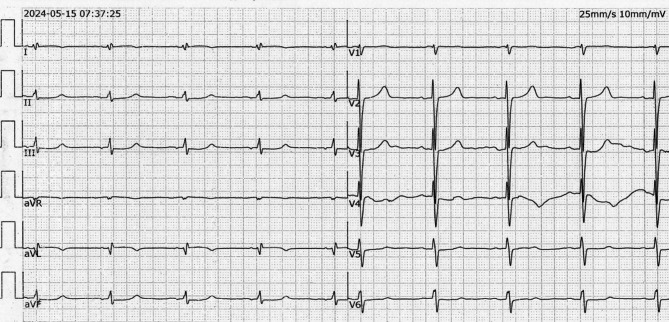
ECG following the removal of the pacemaker leads. The ECG displays a sinus rhythm with a heart rate of 52 beats per minute. There are no episodes of ventricular tachycardia or ventricular premature contractions observed

The patient’s VT resolved, and no further syncope episodes occurred. Postoperative ECG indicated sinus rhythm with a ventricular rate of 50–60 bpm (Fig. 8). The patient was discharged on postoperative day 7 with no recurrence of infection or arrhythmia at follow-up.

## Discussion

This case report presents a unique scenario of a delayed pacemaker pocket infection occurring three years post-implantation, without positive cultures for any specific pathogen. The absence of pathogen growth in blood and local secretion cultures is particularly notable and warrants discussion. Several factors could contribute to this finding [1]. Firstly, the patient’s prior antibiotic treatment at a local hospital before cultures were taken could have suppressed bacterial growth, making it difficult to identify the causative agent. Additionally, the nature of the infection, possibly being subacute or chronic due to the extended period post-implantation, might have led to low bacterial loads that are difficult to culture.

The patient’s profession as a violinist involves repetitive use of the left shoulder, which likely contributed to mechanical wear and erosion at the pacemaker site, increasing the risk of infection. However, the absence of detectable pathogens in the cultures suggests that the inflammatory response could be partially due to non-infectious factors such as mechanical irritation, although the clinical presentation and response to antibiotics indicate an infectious process.

The unique presentation of VT in this case was associated with the pacemaker lead’s positioning in the right ventricular outflow tract, suggesting that the inflammation around the lead, whether infectious or mechanically induced, could exacerbate arrhythmogenicity [2–4]. Although the patient’s ventricular tachycardia (VT) ECG characteristics are not typical, they still suggest a likely origin from the right ventricular outflow tract (RVOT) free wall. The variability in QRS morphology, with lead I showing Rs pattern to rS pattern, leads II, III, aVF showing R pattern, lead V1 showing R pattern, and lead V2 showing rS pattern, may be due to local electrode movement or regional cardiac conduction variations. The termination of VT upon removal of the RVOT free wall electrode further supports this origin despite the atypical ECG presentation [5].

The resolution of symptoms following the removal of the device and administration of antibiotics supports the infectious nature of the inflammation, but also highlights the complexity of diagnosing and treating device-related infections when standard cultures fail to identify a pathogen [6].

The gradual changes in QRS morphology observed during ventricular tachycardia (VT) in this patient with a right ventricular outflow tract (RVOT) pacemaker lead suggest variable contact between the lead tip and myocardial cells. Despite these morphological changes, the VT frequency remains constant, indicating a stable pacing source likely at the lead tip. These observations are independent of body position changes, reinforcing that the QRS variations are due to local contact variations rather than positional changes of the heart. This phenomenon underscores the significant impact of lead tip positioning on QRS waveform characteristics [7–9].

Removing a pacemaker lead three years post-implantation carries a risk of cardiac perforation. However, due to pacemaker pocket and lead infection, removal was necessary. We meticulously prepared for potential complications, including pericardiocentesis and cardiac surgery readiness for emergency open-heart intervention. The extraction of the screw-in lead proceeded smoothly, and the patient experienced no postoperative complications such as pericardial effusion [10–12].

Given the lack of positive cultures, the decision to use broad-spectrum antibiotics was prudent and based on the common pathogens associated with pacemaker infections, primarily Staphylococcus species. This approach is justified in cases where clinical signs of infection are evident, but no specific pathogens are identified, as it covers a broad range of potential bacteria.

The management of the infection through device removal and broad-spectrum antibiotic therapy [13, 14], in this case, was effective, underlining the importance of a comprehensive approach to suspected infections, especially when empirical treatment is necessary. This case emphasizes the need for careful assessment of occupational factors that may compromise implant sites and highlights the challenges of managing infections when culture results are negative [15].

## Conclusion

This case highlights the link between pacemaker infections and occupational habits, and the arrhythmias related to lead positioning. The patient’s violin playing likely caused mechanical stress, leading to infection. Additionally, the ventricular lead’s position contributed to arrhythmias. Effective treatment must consider these factors, including broad-spectrum antibiotics and careful lead placement.

## Data Availability

All relevant data supporting the conclusions of this article are included within the manuscript.

## References

[1] Mela T, McGovern BA, Garan H, Vlahakes GJ, Torchiana DF, Ruskin J, Galvin JM. Long-term infection rates associated with the pectoral versus abdominal approach to cardioverter- defibrillator implants. Am J Cardiol. 2001;88:750–3. 11589841 10.1016/s0002-9149(01)01845-8

[2] Wisoff BG. Pacemaker-Induced Ventricular Tachycardia. JAMA: J Am Med Association. 1965;192. 10.1001/jama.1965.03080160078027 14270292

[3] Atlee JL, Bernstein AD. Runaway Temporary Pacemaker. N Engl J Med. 1980;302:1030–1. 7366611 10.1056/NEJM198005013021813

[4] Bauer A. Imitating ventricular tachycardia. Heart. 2003;89:1382–a. 14617537 10.1136/heart.89.12.1382-a PMC1767989

[5] Landreville JM, Joubert GI, Welisch E, Helleman K, Poonai NP. Atypical presentation of right ventricular outflow tract ventricular tachycardia. J Emerg Med. 2015;49:432–5. 26194529 10.1016/j.jemermed.2014.06.033

[6] DeSimone DC, Sohail MR. Management of bacteremia in patients living with cardiovascular implantable electronic devices. Heart Rhythm. 2016;13:2247–52. 27546815 10.1016/j.hrthm.2016.08.029

[7] Castellanos A Jr., Lemberg L. Cardiac arrhythmias. 6. Pacemaker arrhythmias and electrocardiographic recognition of pacemaker. Circulation. 1973;47:1382–91. 4709550 10.1161/01.cir.47.6.1382

[8] Escher DJ. Types of pacemakers and their complications. Circulation. 1973;47:1119–31. 4574568 10.1161/01.cir.47.5.1119

[9] Erdinler I, Okmen E, Zor U, Zor A, Oguz E, Ketenci B, Akyol A, Aytekin S, Ulufer T. Pacemaker related endocarditis: analysis of seven cases. Jpn Heart J. 2002;43:475–85. 12452305 10.1536/jhj.43.475

[10] Zhang J, He L, Xing Q, Zhou X, Li Y, Zhang L, Lu Y, Tuerhong Z, Yang X, Tang B. Evaluation of safety and feasibility of leadless pacemaker implantation following the removal of an infected pacemaker. Pacing Clin Electrophysiol. 2021;44:1711–6. 34455604 10.1111/pace.14346

[11] Ruttmann E, Hangler HB, Kilo J, Hofer D, Muller LC, Hintringer F, Muller S, Laufer G, Antretter H. Transvenous pacemaker lead removal is safe and effective even in large vegetations: an analysis of 53 cases of pacemaker lead endocarditis. Pacing Clin Electrophysiol. 2006;29:231–6. 16606389 10.1111/j.1540-8159.2006.00328.x

[12] Farooqi FM, Talsania S, Hamid S, Rinaldi CA. Extraction of cardiac rhythm devices: indications, techniques and outcomes for the removal of pacemaker and defibrillator leads. Int J Clin Pract. 2010;64:1140–7. 20642712 10.1111/j.1742-1241.2010.02338.x

[13] Baddour LM, Epstein AE, Erickson CC, Knight BP, Levison ME, Lockhart PB, Masoudi FA, Okum EJ, Wilson WR, Beerman LB, Bolger AF, Estes NAM, Gewitz M, Newburger JW, Schron EB, Taubert KA. Update on Cardiovascular Implantable Electronic device infections and their management. Circulation. 2010;121:458–77. 20048212 10.1161/CIRCULATIONAHA.109.192665

[14] Ben Abid F, Al-Saoub H, Howadi F, AlBishawi A, Thapur M. Delayed Pacemaker Generator Pocket and lead primary infection due to Burkholderia Cepacia. Am J Case Rep. 2017;18:855–8. 28769025 10.12659/AJCR.904435 PMC5551955

[15] Sohail MR, Uslan DZ, Khan AH, Friedman PA, Hayes DL, Wilson WR, Steckelberg JM, Stoner S, Baddour LM. Management and outcome of Permanent Pacemaker and Implantable Cardioverter-Defibrillator infections. J Am Coll Cardiol. 2007;49:1851–9. 17481444 10.1016/j.jacc.2007.01.072

